# How the Digital Product Passport Can Lead the Plastics Industry towards a Circular Economy—A Case Study from Bottle Caps to Frisbees

**DOI:** 10.3390/polym16101420

**Published:** 2024-05-16

**Authors:** Thomas Rumetshofer, Klaus Straka, Jörg Fischer

**Affiliations:** 1 Institute of Polymeric Materials and Testing, Johannes Kepler University Linz, Altenberger Straße 69, 4040 Linz, Austria; 2 Institute for Polymer Injection Moulding and Process Automation, Johannes Kepler University, Altenberger Straße 69, 4040 Linz, Austria

**Keywords:** digitalization, traceability, circular economy, Digital Product Passport, sustainability, plastics recycling

## Abstract

The Digital Product Passport (DPP) as a product-specific data set is a powerful tool that provides information on the origin or composition of products and increases transparency and traceability. This recycling case study accompanies the production of 2192 frisbees, which originated from collected beverage bottle caps. In total, 486.7 kg of feedstock was collected and transformed into 363.2 kg of final product with verified traceability through all process steps via a DPP, provided by the R-Cycle initiative and based on the GS1 standard. This demanded a generally agreed dataset, the availability of technical infrastructure, and additional effort in the processing steps to collect and process the data. R-Cycle offers a one-layer DPP where the data structure is lean and information is visible to everyone. This is beneficial to a variety of stakeholders in terms of transparency. However, it does not allow the sharing of sensitive information. On the one hand, the DPP has a high potential to be an enabler for customer engagement, origin verification, or as a starting point for more efficient and advanced recycling of plastics. On the other hand, the DPP involves a certain effort in data generation and handling, which must be justified by the benefits. For small, simple packaging items, the DPP may not be the perfect solution for all problems. However, with a broader societal mindset and legislative push, the DPP can become a widely used and trusted declaration tool. This can support the plastics industry in its journey towards a circular economy.

## 1. Introduction

In 2020, the global production of plastics, excluding recycled plastics, was 367 Mt, of which 55 Mt came from European production [1]. At the same time, approximately 30 Mt of post-consumer waste was collected in Europe, of which about 35% was sent to recycling facilities inside and outside Europe, 42% to energy recovery facilities, and still around 23% to landfills. The installed recycling capacity in Europe reached 8.5 Mt in 2020, with polyethylene terephthalate (PET) accounting for a share of around 30% [2]. Geyer et al. [3] calculated that the cumulative production of resins and fibers manufactured from 1950 to 2015 was about 8300 Mt, generating approximately 6300 Mt of plastic waste in the same period.

The European legislation recognized this problem and adopted the Circular Economy Action Plan 2020 to focus on sustainability along the entire life cycle of products [4]. The aim of this action plan is to make sustainable products the norm in the EU and to empower consumers in their choice, focusing on batteries, vehicles, packaging, plastics, and textiles, where the potential for circularity is high. Since 2009, the Ecodesign Directive [5] has provided a framework for product reusability, reparability, energy efficiency, resource efficiency, recycled content, environmental footprint, and information requirements for energy-related products. This includes the introduction of a product passport or Digital Product Passport for better verification and control. In 2022, a proposal was announced to extend the existing Ecodesign framework to the widest possible range of products and to broaden the scope of requirements [6]. This proposal now includes plastics, packaging, textiles, and construction and extends the requirements that all regulated products will have a Digital Product Passport (DPP), which focuses on the final product characteristics and is mainly intended for customer engagement. However, its ability to act as a proof of origin or as a source of information for more efficient and advanced recycling of plastics is an additional benefit. King et al. [7] extracted the requirements and perspectives from European proposals and identified a universal model for a DPP based on nine high-level capabilities to ensure interoperability across multiple product life cycles, organizations, supply chains, and value chains. In addition to technical advancements, a DPP and traceability offer potentially new possibilities for business models linked to the circular economy of plastic materials [8].

As threats to all digital solutions, data security, leakage of data, authenticity of data or partners, fault detection, value chain security, and others are frequently mentioned [9,10]. Pennekamp et al. [11] investigated data flows and privacy perspectives in interconnected production networks and concluded that there is currently no one-size-fits-all solution for all privacy and security challenges available. Hiller et al. [12] explored a tailored onion routing protocol for untrusted mobile networks to provide anonymity and protection against attacks. Duy et al. [13] propose a federated multivariate statistical process control (FedMSPC) framework for material and process data sharing across company borders to ensure privacy-preserving transfer and anonymity of shared content. While all of these researchers presented solutions for providing data along the process chain, an example of implementing a DPP in the plastics circular economy is still missing.

The main objective of this case study was to investigate the recyclability of post-consumer beverage bottle caps and demonstrate traceability along the recycling process from waste to final product through the practical implementation of a DPP. The focus of this paper is to demonstrate traceability by including all production and process data in the R-Cycle initiative database. This research digitally follows the path of the material from cap to frisbee and discusses the benefits of a DPP. Akhras et al. [14]. confirmed in an earlier study the recyclability of this process by performing various mechanical and chemical analyses.

## 2. Digital Product Passport

A Digital Product Passport (DPP) is defined as a product-specific data set that can be accessed electronically via a data carrier and that can provide information on the origin, composition, recycling options, repair possibilities of a product, and much more [15]. The introduction of a DPP promises a number of benefits, such as access to information for consumers and policymakers, increased transparency, traceability and consistency of data, source for the development of entirely new business models, and optimization of resources and energy flows. To minimize effort, the number of data points collected and the quality of the data are influential parameters.

Essential components for a well-functioning DPP are the ways of data exploitation (tagging of products), the choice of database used, efficient ways of data curation (data denoising and data cleaning), data sharing concepts, and finally the leverage of all collected data [16]. Plociennik et al. [17] identified information requirements, collaboration along the value chain, product identification, and fulfillment of legal obligations as the main requirements for establishing a DPP. In addition to data for production and recycling, a DPP can contribute to customer engagement by storing and accessing user-friendly information, such as user manuals, assembly instructions, performance statistics, material origin, circularity indicators, environmental impact indicators, recycling information, and others [18]. Jensen et al. [19] investigated critical decision points and data needs for DPP in the context of mechatronics and highlighted the important role of policymakers and DPP as a support tool for decision-making. Although many independent developments of DPP are underway, some common drawbacks of DPP remain. Diverging interests of stakeholders, lack of standardization, lack of infrastructure, unresolved data ownership, and lack of clear rules on how to handle individual know-how and intellectual property rights (IPR) can be mentioned as obstacles [15].

Voulgaridis et al. [16] propose a cloud-based digital passport version consisting of a two-layer structure, Passport Generation and Digital Passport, which allows the separation of business-sensitive and consumer-relevant data. In the Passport Generation phase, all production data from different business partners are provided and collected separately in a closed system to increase data security and trustworthiness. In the second phase, a Digital Passport is created to interact and engage with customers. Plociennik et al. [20] propose a cloud-based Digital Lifecycle Passport (DLCP), consisting of a combined digital lifecycle passport and an asset administrative shell (AAS) part, to enable a better recycling process and generate a robust lifecycle assessment. The AAS is a digital abstraction of the asset used to manufacture a product and represents all physical or non-physical asset information for modeling the product lifecycle, while the DLCP is filled with all data generated during the production processes. To overcome the challenges of data evaluation for circularity, Mulhall et al. [21] recommend a Product Circularity Data Sheet (PCDS) as a globalized open-source industry standard that contains basic circularity data about products and their ingredients.

### 2.1. Digital Product Passport from R-Cycle

Based on the GS1 open standard, R-Cycle provides a data infrastructure for operating a DPP for plastic products that is able to link physical products to digital data [22]. The core of the R-Cycle technology is data infrastructure that can link events along the value chain, ensure proper data management, transfer information from one stakeholder to another, help increase efficiency along the value chain, and build customer trust through full traceability. Another anchor point is the flexibility of data usage alternatives as a bridge between physical products and the digital data stream, ranging from a simple barcode to more advanced techniques such as digital watermarks. To support data sharing within the packaging industry, GS1 released a standard on circular plastic traceability, which defines 24 different data attributes and shows examples of different events [23].

### 2.2. EPCIS and GTIN Standard

The Electronic Product Code Information Services (EPCIS) protocol is an important international standard, defined by ISO/IEC 19987 to create, store, share, and visualize event data of physical or digital objects across company borders and is provided by GS1 [24]. This standard explains the different layers of the data model, defines the capture interface and the query interface, and adopts a hierarchical, modular, and scalable design [25]. Nowadays, EPCIS is being used to increase transparency, quality, efficiency, and control within supply chains, such as temperature control in cool chains or to verify the origin of meat and fish [26,27]. Li et al. [28] propose EPCIS as an extension to a blockchain-based traceability system to implement location discovery and improve interactivity.

Identification of products in the grocery store is realized by the Global Trade Item Number (GTIN), covered in the product bar code, and provided by GS1 [29]. For decades, this system has been used for the identification of products on different hierarchy levels and uses a unique reference number as a key for identification. Without further information, GTIN is not able to track a single item and retrieve information on place and time.

## 3. Methodology

Existing DPP systems for traceability are not optimized for the plastics industry or the inclusion of recycled materials. Within this work, we aim to find optimized solutions for plastic recycling and to develop digital solutions with broad acceptance in the industry. After finding a common understanding of the recycling process steps, R-Cycle chose EPCIS as a standardized protocol and unified language to communicate process event data in five different dimensions [30]. In addition to object, location, and time, information related to business context and state can be transferred for each individual predefined process step. In R-Cycle, a DPP is available for each process step and can be used for supplier and customer engagement. The final DPP contains individual datasets of information that work together to provide a complete tracking history. The datasets had to be negotiated between all parties in the recycling process, and within the categories, they are divided into mandatory fields, which are agreed upon by all stakeholders along the value chain to ensure meaningful results, and voluntary fields, which are optional and can be shared to increase the quality and acceptance of the DPP. 

To ensure traceability of individual events within the R-Cycle environment, lot-based Global Trade Item Numbers (LGTIN) are provided that contain, in addition, information on business location and time [29]. This number represents a unique, traceable identification for each lot along the value chain and is provided by GS1.

The templates for the following case study were developed in several workshops and represent what information is needed to process a mechanical recycling process from bottle caps to a final product. At the same time, certain types of sensitive information were excluded from the R-Cycle framework to prevent leakage of trade secrets or business-critical information. This is a trade-off between the sharing of information to guarantee transparency and the protection of secrets that needs to be defined for every application separately. In the following section, the adapted protocols for R-Cycle are applied to the use case study from bottle caps to frisbees.

## 4. Results and Discussion

### 4.1. Case Study: From Bottle Caps to Frisbees

**Figure 1:**
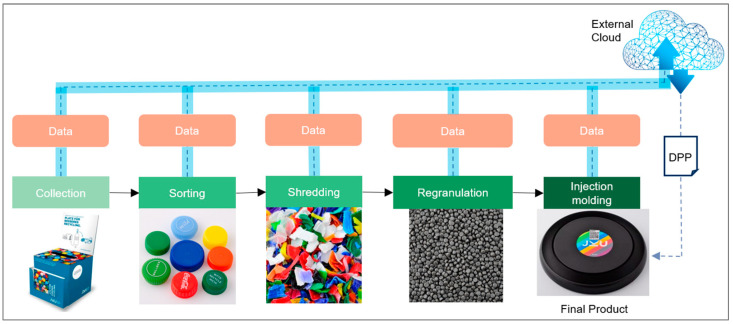
Performed process steps from collection to the final product for this case study [31,32].

**Figure 2:**
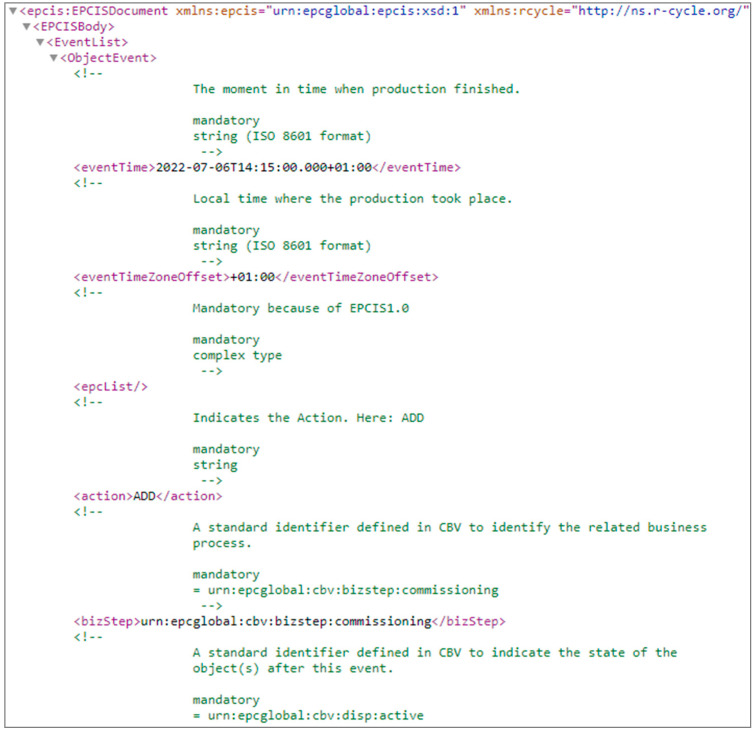
Partial example of Python code for the transfer of information [31].

To demonstrate the traceability and use of a DPP in an open-loop plastics recycling process, a case study was conducted at the LIT Factory (Linz, Austria) to convert high-density polyethylene (HDPE) bottle caps into a frisbee disc end product [31]. Figure 1 illustrates the process steps from collection to end product. In addition to the material flow at each process step, information as a central element was created, collected, and communicated as a unique LGTIN for each event. For each process step, a predefined set of parameters was required and communicated to R-Cycle. The data for the DPP generation are stored on an external server and the data transfer per event was realized via a predefined XML file coded in Python. Figure 2 shows exemplary a part of the code for the transfer of data to R-Cycle.

**Figure 3:**
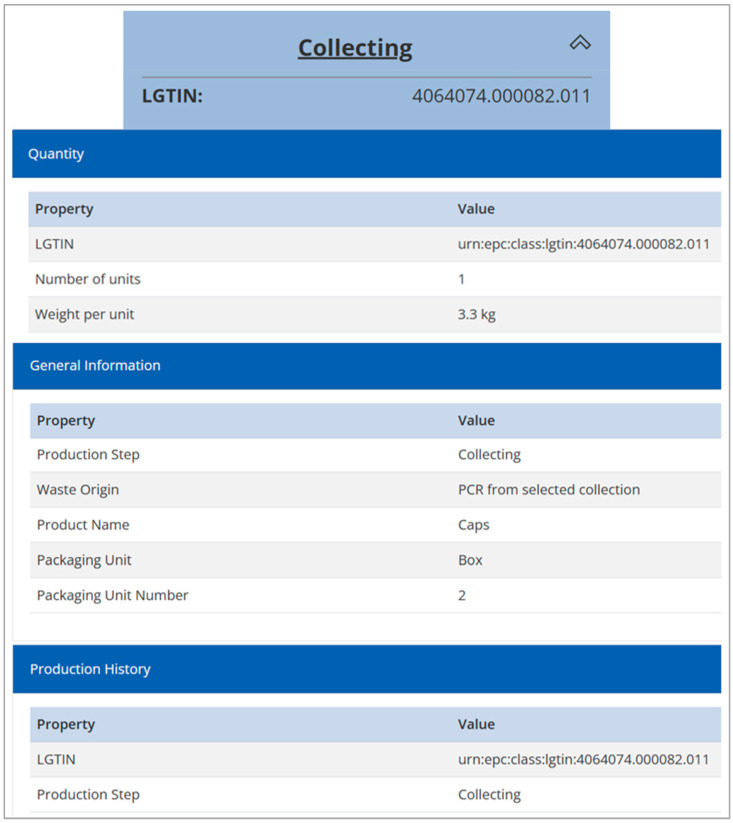
Example of product data and product tree for the collection step of Lot 11 on the R-Cycle platform, reprinted from [31].

An additional goal of this demonstration project was to raise awareness of the circular economy among young people. Therefore, a collection competition was launched in schools and other educational institutions in Upper Austria with 19 participants and 103 collection boxes distributed, resulting in a material yield of about 251 kg. To ensure the operation of a mechanical recycling process on a semi-industrial scale, an additional 235 kg of HDPE bottle caps from separate collections were provided by LAVU (OÖ. Landes-Abfallverwertungsunternehmen GmbH, Wels, Austria). For each participant, a separate collection event represented by a unique LGTIN was created, and the necessary data and information in three different categories were collected, transferred to the R-Cycle server, and used as input for subsequent process steps [31]. Figure 3 shows the product data and product tree for the Lot 11 collection as an exemplary dataset.

**Figure 4:**
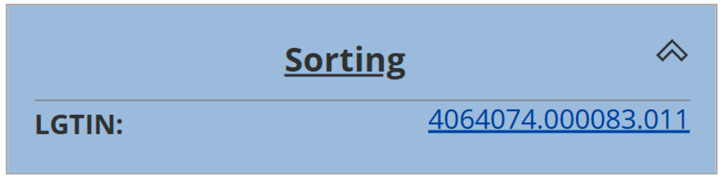
Product tree for Lot 11 as an example of the sorting step on the R-Cycle platform, reprinted from [31].

The second process step was to manually sort the collected material. During this process step, non-polymeric materials, caps with labels, caps from non-beverage applications, and caps clearly identified as non-HDPE material were removed by hand. All relevant data for further processing were documented. In total, approximately 10% of the collected material stream was removed at this stage. No specific color sorting step was performed, resulting in a mixture of all colors supplied in the final recyclates. Figure 4 shows an exemplary product tree for Lot 11 and Table 1 shows the process data collected during the sorting step for Lot 11, as well as the corresponding data from the collection step, to demonstrate that some data are partly transferred from one process step to the next [31]. Some business-critical properties within the DPP are only exchanged in encrypted format to ensure security. The key is generated and provided by the R-Cycle initiative. In our example, the location and machine ID are only shared in encrypted form.

**Figure 5:**
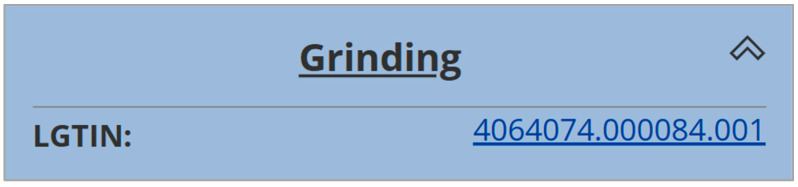
Product tree for the grinding step on the R-Cycle platform, reprinted from [31].

As a third process step, the sorted bottle caps were shredded into flakes on a Lindner Micromat 1500 industrial single-shaft shredder located at LIT Factory in Linz, Austria. All relevant process data were collected in five different categories, a unique LGTIN was created, and data were provided for DPP creation. Figure 5 shows the product tree for this grinding step and Table 2 presents the corresponding product data [31]. At this stage, information from the sorting step of all 20 batches is combined into one dataset for the grinding step.

**Figure 6:**
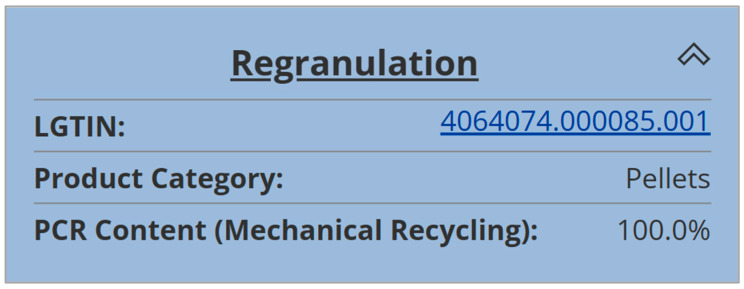
Product tree for the regranulation step on the R-Cycle platform, reprinted from [31].

In the fourth process step, the flakes were converted into HDPE recyclates on an INTAREMA 1108 TVEplus recycling extrusion line from the Erema Group. This line is equipped with filtration and degassing systems and is also located at the LIT Factory in Linz, Austria. All relevant quality and process data of this step were collected in five different categories in the local data infrastructure of the LIT Factory and provided for the DPP creation. Figure 6 depicts the product tree and Table 3 provides the product data for the regranulation step [31].

To evaluate the product quality, after the regranulation step, samples were taken, prepared, analyzed, and reported by Akhras et al. [14] using different analytical and mechanical tests. A part of these results is presented in the product data table as voluntary information to increase the value of the finally produced DPP. If more quality data are available, it can be added additionally to the respective DPP.

**Figure 7:**
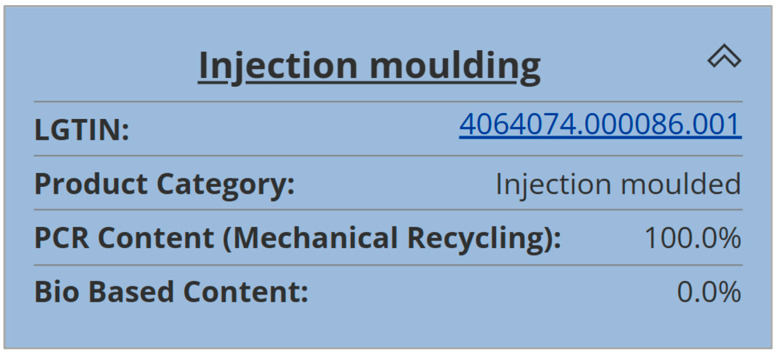
Product tree for the injection molding step on the R-Cycle platform, reprinted from [31].

In the final process step, the end product was injection molded on an Engel Duo 350 injection molding machine. In total, 2192 frisbees were produced. In this step, the relevant process data were collected in five different categories, stored locally, and transferred via XML file for DPP creation. Figure 7 illustrates the product tree and Table 4 presents the product data for the injection molding step [31].

**Figure 8:**
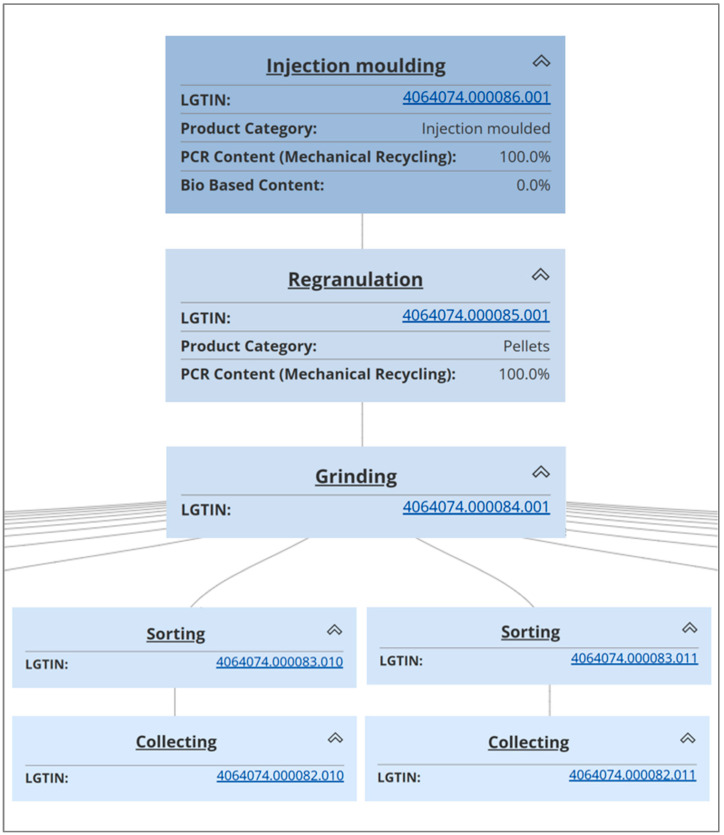
Product tree for the final DPP of the case study, reprinted from [31].

All process steps are linked and summarized to build the final product tree for the DPP on a batch basis, as shown in Figure 8. The information in the product data table was either generated within each process step or transferred from previous steps. The DPP could be much more comprehensive if additional quality data from laboratory or feedstock sources were included within existing process steps. In the R-Cycle initiative framework, an extension of process steps is only possible with the agreement of all parties along the value chain.

### 4.2. Use Case: Mass Balance Based on DPP Data

**Figure 9:**
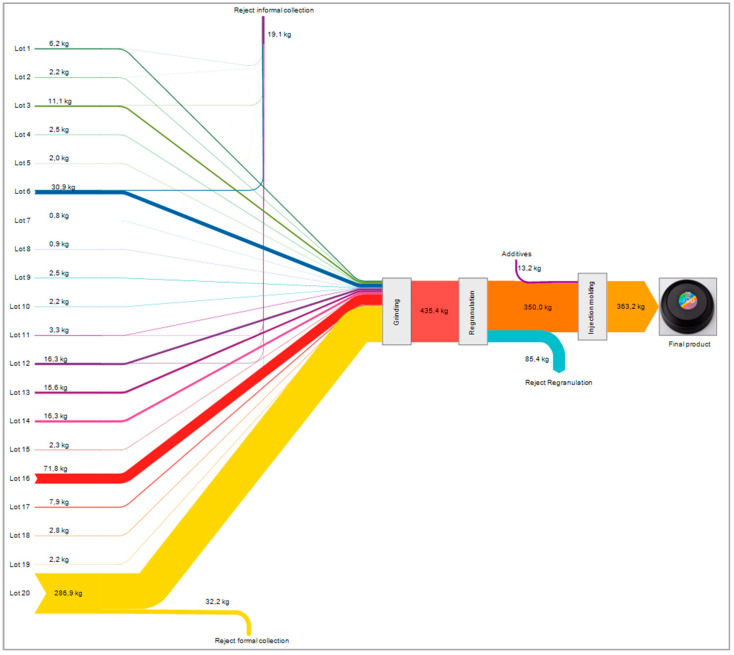
Mass balance for final DPP data of this case study as Sankey diagram [31].

A major advantage of storing data in a DPP is that all information along the production chain is combined and accessible in one spot. These data points can be used for many different purposes. For example, the DPP data can be used to create a complete and comprehensive mass balance from collection to the final product. Figure 9 illustrates the full mass balance for the described case study as a flow diagram over all process steps using e!Sankey software Version 5.2.1 (Rev. 0). A DPP offers the advantage of closed system boundaries for the product or batch and allows verification of data. In this way, customers can trust the verified data that are not directly accessible. In addition to mass balance, many other applications with comprehensive data are possible, like energy demand, product quality, or product origin.

## 5. Conclusions

As shown in this recycling case study of a frisbee production from high-density polyethylene bottle caps, traceability through all process steps via a Digital Product Passport (DPP) is already possible for all 2192 end products based on the established GS1 structure. This requires the availability of technical requirements and additional effort in some process steps to collect and process the data. Especially where manual handling is involved, data must be manually documented and manually transferred to the appropriate DPP database. Wherever process data can be generated and transferred automatically, the effort is reduced. In the case of R-Cycle, a one-layer DPP is used, where the data structure is lean and information is visible to everyone, which is favorable for transparency but does not allow the sharing of sensitive information. The final DPP of the frisbee can then be used for many different practical purposes.

Verification of product or ingredient origin and its authentication to generate trust within the value chain or towards customers is a relevant topic and has become more important in a globalized world. In the food industry, verification is investigated for region authentication quality insurance and requires a sophisticated accreditation system or heavy analytical capabilities [34]. For plastic materials, an accredited DPP could solve or support verification of regional origin.

The mechanical recycling process for plastics is heavily influenced by contamination, foreign polymers, or harmful substances [35]. Having more information on composition, quality, and harmful components can significantly improve the quality of the final recyclates material and widen the possible applications due to increased trust. A comprehensive DPP can provide this information from one recycling cycle to the next.

In today´s changing business environment, service improvement and customer experience management are crucial success factors [36]. Considering the additional requirement of data collection and management and the corresponding efforts, proper applications and additional information for DPP must be found. This can range from a paperless user instruction to the origin of the food we buy every day at the supermarket.

DPP comes with a certain amount of effort in terms of data generation and handling, which needs to be justified by its benefits. Hence, a common understanding of the meaning and content of DPP in the scientific community and public opinion is needed. In the language of legislation, DPP focuses on the end product and the customer, while in the R-Cycle DPP, relevant data for each process step are available for all players within the supply chain. Another important point is the data quality of all entered data points, which influences the usability and quality of all subsequent usage. Even in this frisbee use case, the time-stamp data of some process steps was not entered or transferred in the correct way, and therefore no time correlation between the steps can be completed. This emphasizes the quality of entered data as well as the development and implementation of measures to check the quality and consistency of added data points.

With a broader mindset in society and legislative push, DPP can become a widely used and accepted tool for trustworthy declaration. This can support the plastics industry on its way to a circular economy.

## Figures and Tables

**Table 1:** Table with product data for the collection and sorting steps of Lot 11 on the R-Cycle platform, reprinted from [16].

Property	Value Collecting	Value Sorting
**Quantity**		
LGTIN	urn:epc:class:lgtin:4064074. 000082.011	urn:epc:class:lgtin:4064074. 000083.011
Number of units	1	1
Weight per unit	3.3 kg	2.6 kg
**General information**		
Production Step	Collecting	Sorting
Waste Origin	PCR from selected collection	PCR from selected collection
Product Name	Caps	Caps
Product Class		PE
Packaging Unit	Box	Box
Packaging Unit Number	1	1
**Production History**		
LGTIN	urn:epc:class:lgtin:4064074. 000082.011	urn:epc:class:lgtin:4064074. 000083.011
Production Step	Collecting	Sorting
Business Location		urn:epc:id:sign:406407400080.0
Machine ID		urn:epc:id:sign:406407400080.0
Internal Machine ID		None
Internal Machine Type		Manual

**Table 2:** Table with product data for the grinding step on the R-Cycle platform, reprinted from [16].

	Property	Value
**Quantity**		
	LGTIN	urn:epc:class:lgtin:4064074.000084.001
	Number of units	1
**General information**		
	Production Step	Grinding
	Waste Origin	PCR from selected collection
	Product Name	Flakes
	Product Class	PE
	Packaging Unit	BigBag
	Packaging Unit Number	1
**Basic Properties**		
	Maximum Particle Size	10.0 mm—Norm: None
**Ingredients**		
	Ingredient List	PE—100.0%
**Production History**		
	LGTIN	urn:epc:class:lgtin:4064074.000084.001
	Production Step	Grinding
	Business Location	urn:epc:id:sign:4064074.00080.0
	Machine ID	urn:epc:id:sign:4064074.00080.2
	Internal Machine ID	LM-1
	Internal Machine Type	Lindner Micromat

**Table 3:** Table with product data for the regranulation step on the R-Cycle platform, reprinted from [16].

	Property		Value
**Quantity**			
	LGTIN		urn:epc:class:lgtin:4064074.000085.001
	Number of units		1
	Weight per unit		350.0 kg
**General information**			
	Production Step		Regranulation
	Product Category		Pellets
	Waste Origin		PCR from selected collection
	Product Type		Polymer
	Recommended Use		
	Product Name		None
	Product Class		PE
	Packaging Unit		BigBag
	Packaging Unit Number		1
**Basic Properties**			
	MFR	Value	4.18 g
		Condition	/10 min @ 190 °C 5 kg
		Norm	DIN EN ISO 1133-1 [33]
	Color Note		Multicolored
	Color Lab	L-Value	34.532
		a-Value	0.47
		b-Value	−0.51
		Condition	-
	Filtration Fineness		90 µm
**Ingredients**			
	Ingredient List		PE—100.0%
	PCR Content (Mechanical Recycling)		100.0%
**Production History**			
	LGTIN		urn:epc:class:lgtin:4064074.000085.001
	Production Step		Regranulation
	Business Location		urn:epc:id:sign:4064074.00080.0
	Machine ID		urn:epc:id:sign:4064074.00080.3
	Internal Machine ID		P-20180262
	Internal Machine Type		Recycling Extruder

**Table 4:** Table with product data for the injection molding step on the R-Cycle platform, reprinted from [16].

	Property	Value
**Quantity**		
	LGTIN	urn:epc:class:lgtin:4064074.000086.001
	Number of units	2192
**General information**		
	Production Step	Injection molding
	Product Category	Injection molding
	Product Type	Polymer
	Product Sector	Technical
	Product Name	JKU LIT Factory Frisbee
**Basic Properties**		
	Part Weight	165.7 g
	Density	0.949 g/cm^3^
**Advanced Properties**		
	Outside Surface Type	PE-HD
**Ingredients**		
	Ingredient List	
	MFR Range	PE—4.0–10.0 g/10 min @2.16 kg, 190 °C
	PCR Content	100.0%
	Bio-based Content	0.0%
**Production History**		
	LGTIN	urn:epc:class:lgtin:4064074.000086.001
	Production Step	Injection molding
	Business Location	urn:epc:id:sign:406407400080.0
	Machine ID	urn:epc:id:sign:406407400080.1
	Internal Machine ID	224759
	Internal Machine Type	ENGEL DUO2460/350

## Data Availability

The raw data supporting the conclusions of this article will be made available by the authors on request.
